# Locate your soundscape: interacting with the acoustic environment

**DOI:** 10.1007/s11042-021-10683-9

**Published:** 2021-03-25

**Authors:** Nicola Orio, Berardina De Carolis, Francesco Liotard

**Affiliations:** 1 Department of Cultural Heritage, University of Padua, Piazza Capitaniato, 7, Padua, Italy; 2 Department of Information Technology, University of Bari “Aldo Moro”, Via E. Orabona, 4, Bari, Italy; 3 Moovioole s.r.l, Via don Giuseppe Rizzi, 26, Bassano del Grappa (VI), Italy

**Keywords:** Soundscape, Mobile app, Intangible heritage, Crowdsourcing

## Abstract

Although overshadowed by visual information, sound plays a central role in how people perceive an environment. The effect of a landscape is enriched by its *soundscape*, that is, the stratification of all the acoustic sources that, often unconsciously, are heard. This paper presents a framework for archiving, browsing, and accessing soundscapes, either remotely or on-site. The framework is based on two main components: a web-based interface to upload and search the recordings of an acoustic environment, enriched by in- formation about geolocation, timing, and context of the recording; and a mobile app to browse and listen to the recordings, using an interactive map or GPS information. To populate the archive, we launched two crowdsourcing initiatives. An initial experiment examined the city of Padua’s soundscape through the participation of a group of undergraduate students. A broader experiment, which was proposed to all people in Italy, aimed at tracking how the nationwide COVID-19 lockdown was dramatically changing the soundscape of the entire country.

## Introduction

Cultural heritage is a broad term that refers to both tangible and intangible human artifacts. It also includes natural elements relevant to a given place or society. Although these components can, in principle, be discerned by all sensory channels, most attention is usually paid to tangible artifacts that are visually perceived. Consequently, the application of information technologies to the dissemination of cultural heritage has privileged visual content. The role of music as a significant contributor to cultural heritage is undisputed, and there are numerous projects regarding the relationship between music and places, from the use of music as an aid to teaching geography [31] to the automatic identification of music that is representative of a geographical area through social media mining [24]. Musical works have been geolocated and made accessible through a web interface representing a stylized planisphere [19] and cartography inspired *Island of Music* [23], a two-dimensional representation of a music collection obtained from self-organizing maps.

Yet, with the obvious exception of speech, non-musical audio has received relatively little attention in cultural heritage applications, despite sound being powerfully evocative of populations, places and historical periods. The automatic analysis of the acoustic environment, called acoustic scene classification [3], focuses on methods for assessing the acoustic quality of a location [13] rather than its cultural aspects. More recently, several projects are beginning to examine the auditory channel, measuring its effectiveness in exploring an environment [34], navigating a tourist map [20] and giving auditory feedback to visitors of cultural sites [25].

This paper reports on a project concerning the recording, archiving, searching and enjoyment of acoustic environments, or *soundscapes*. The paper is organized as follows: The first part introduces the concept of soundscapes in Section 2 and then examines several relevant related works in Section 3. Section 4 of the second part describes the different components of the *Locate Your Sound* system. These are responsible for uploading and archiving soundscapes, navigating or searching the collection using a web interface, interacting with the acoustic environment of a site through a mobile app and smart panels, and for accessing the recorded soundscapes made in the user’s location while on the move. Section 5 discusses two crowdsourcing initiatives to populate the system. Indeed, Locate Your Sound is currently being used to track and record how the nationwide lockdown has changed the acoustic environment. The effectiveness of the approach is discussed in Section 6. Section 7 presents several conclusions.

## Soundscape

The term *soundscape* refers to the acoustic environment that characterizes and defines a location with unique features to the audio channel. First of all, sound perception is closely related to time. Not only is sound perceived as temporal variations of pressure waves but, since there is an alternation and a succession of different acoustic sources in many environments, sound also has an intrinsic *narrative* component. Moreover, the same location may have entirely different soundscapes depending on the time of day or the year. For instance, at noon in the square in front of the Padua Cathedral, the most prominent sound comes from the church bells, the acoustic environment in the early afternoon of a weekday is characterized by the high pitched noises of children coming home from school and, some hours later, it is mostly created by groups of young adults gathering for happy hour at local bars [22]. As another example, the soundscape of a tourist destination dramatically changes during the off-season due to fewer people or different weather conditions. The changes over time of acoustic sources can also be the starting point for an immersive virtual reality environment that evokes a society of the past to enhance the users’ experience while visiting an archaeological site [29] or to provide an immersive environment for a serious game on historical sites [2].

This diachrony of acoustic sources (i.e., their evolution over time) is enriched by their synchronicity (i.e., the fact that certain events happen simultaneously). In the case of simultaneous events, some sources usually capture a listener’s attention while others remain in the background. By listening carefully, it is possible to deconstruct all these layers of sound and focus on individual sources. It has been proposed [6] that synchronous sounds are perceived through *attentive listening*, which is related to identifying acoustic sources and their characteristics, and through *holistic listening*, related to the general impression that the soundscape produces. They both contribute to the effect of a soundscape, whether the person is seeking out specific, anticipated events, following a story within a multitude of acoustic stimuli, or merely immersed in the environment. Although it is impossible to avoid hearing sound as it is immersive, audio perception is subject to auditory masking: loud sounds capture a listener’s attention and let softer sounds go unnoticed. Again, time is important as the length of a soundscape recording may help a listener detect subtle layers. However, there is no consensus on the time-scale of soundscapes, which are considered relevant when they span a few seconds [5] or when ranging from minutes to hours [29].

A way to classify soundscapes is related to the balance between natural and man-related events [17], with a focus on recognizing the acoustic source:

- **Earth**: e.g., wind, sea waves, waterfalls.
- **Animal**: e.g., screams, calls, chirps.
- **Human**: e.g., the voice of one or more persons, crowd noise, footsteps, claps, breath.
- **Machinery**: e.g., engines, alarms, mechanical parts in movement.

**Fig. 1:**
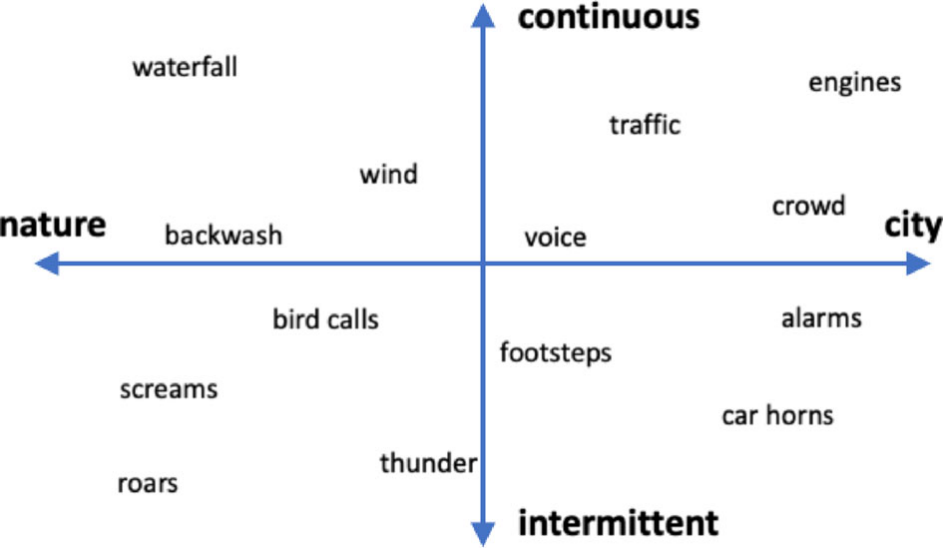
A two-dimensional mapping of different acoustic sources, depending on the type of location (nature vs. city) and the type of sound (intermittent vs. continuous)

Another classification approach is based on the estimate of the location, from natural to urban environments, and on the characteristic of the audio signal, from intermittent/impulsive to stationary/continuous. According to this approach, acoustic sources can be mapped on a two-dimensional graph, as shown in Fig. 1.

Soundscape research began as a part of projects on environmental health, with a focus on potential unease in urban environments, e.g., due to the noise produced by machinery [30]. It evolved into an interdisciplinary research area where sound is considered a resource rather than a pollutant; the focus moved towards considering preference rather than discomfort [8]. As a contribution to the formalization of the discipline, the COST TUD Action “Soundscape of European Cities and Landscapes” [15] investigated issues related to the definition of soundscapes; how they can be measured, represented and designed; and their relationship to the quality of life, the cultural heritage and the history of locations.

A survey [26] carried out in 2017 found that 28 mobile soundscape-related apps were developed between 2008 and 2016. More than half were noise meters, while the rest allowed users to record audio and add user feedback. This survey also highlighted the duality between noise-controlling approaches and soundscape approaches.

## Related work

Soundscapes have been the subject of research by composers, musicologists, and environmentalists since the seventies when the Canadian composer R.M. Schafer popularized the term [28]. In particular, B. Truax theorized how environmental sound could be introduced as an element of the process of composing [33]. Because of its effect on how a location is perceived, soundscape has been used in several installations. As an example, the city of London became the location of an interactive installation where the public environment was enriched by acoustic cues triggered by user movements in the urban space [1].

Soundscape research has been applied to tourism [10] and the enjoyment of cultural heritage. As previously mentioned, visitors’ experience can be enriched by the recreation of sounds that augment the actual environment with evocative or informative cues. Audio augmented reality can be used to let visitors hear sounds as if produced by old playback machinery, bridging the gap between audio and artifact archives [9]. War-related sounds have been used to attract visitors to given locations and evoke impressions in a site with the remnants of trenches and fortified camps from World War I [25]. Sound has been used as the leading cue in a serious game developed to engage visitors of an archaeological park [2, 32]. The effectiveness of audio augmented reality has been analyzed during a test with several visitors of an archaeological site, showing enhanced stimulation in contrast to a control group [29]. This being said, the effect of synthetic soundscapes must be studied in greater detail because they may go unnoticed when presented with visual cues [11].

The influence of involving users in creating an artificial soundscape has also been investigated [27]. In this case, museum visitors took an active role in generating a chosen location’s soundscape by creating a mix from a number of available audio files. Results with a group of students have shown sounds’ ability to evoke experiences and emotions rather than memories. An alternative approach to synthetic soundscapes, centered around cultural landmarks, has been tested to provide visitors with audio cues that improve their wayfinding within a cultural site [20].

In the case of real soundscapes, data gathering is an important issue because the extensive recording of an area requires an enormous amount of time and effort. One solution involves creating a sound sensor network where a number of devices provided with a microphone communicate their input to a central database through the Internet. This approach has been proposed to track an indoor environment’s noisiness using custom-built devices [35] or to classify different sound typologies in a city using the microphones of people’s mobile devices through an app working in the background [30]. Another solution can be based on crowdsourcing, that is, on the voluntary participation of final users who record and upload their own recordings. For instance, the *Soundsslike* project [36] invited users to upload their recording of the city of Istanbul on a web interface and promoted the initiative through an interactive installation. Other crowdsourcing approaches are seen in the projects *Record the Earth* [14] and *Participatory Soundscape Sensing* [18]. In both projects, participants are invited to use a mobile app to record and upload their recordings from all over the world; data is then analyzed and classified according to the acoustic source. Although it is not focused on soundscapes, *Freesound* [12] is another online audio platform, which includes environmental sounds; it is noteworthy that this platform is aimed at audio experts and many recordings are of professional quality and have been used as incidental sounds in movies. Soundscape data collection has also been the subject of specific projects [16] where both quantitative (e.g., sounds) and qualitative (e.g., descriptions and impressions) data were collected from an urban environment and represented in space and time using direct access or animations.

Soundscape representation is generally based on interactive two-dimensional maps. Depending on the application context, the map may be: an artistic elaboration of a city plan [1, 16], a representation of an ancient map [2], a virtual reality environment [11], a geographic map [14], a satellite view [29], a cadastral map [35], and even a multitouch screen [7]. Thematic maps have also been made [36] using categories based on location type. The effectiveness of soundscape representations has been studied [21], focusing on their applications on natural human-computer interaction.

## Locate Your Sound

This paper introduces *Locate Your Sound* (LYS), a novel system dedicated to real soundscapes, where acoustic data is gathered through crowdsourcing. One crucial characteristic of LYS is that user involvement is promoted through regular thematic initiatives involving public and private organizations interested in the preservation and dissemination of intangible cultural heritage. The basic idea is that pure recording of acoustic environments is appealing only for a limited number of audiophiles. In contrast, participation in a focused campaign with a clear goal – even if not directly aimed at acoustic applications – can engage larger groups. To this end, LYS promoted campaigns aimed at ethnographic documentaries, film schools, tourism, and social changes. The latter two are described in this paper. User-generated recordings belonging to different campaigns are organized as separate layers on the same geographic map. Thus, the same physical location can be described by different soundscapes, which is a fundamental characteristic of soundscapes as described in Section 2.

The design and development of LYS were guided by three main goals:

- Documenting the acoustic environment as a fundamental element of the intangible cultural heritage.
- Assessing the effectiveness of soundscapes in describing locations to promote tourism.
- Fostering awareness towards sound and its impact on how the environment is perceived.

In order to achieve these goals, we envisaged different ways to access soundscapes. All functions can be activated through a standard approach based on searching and browsing a web site, which provides an interface for faceted search and a digital map labeled with soundscapes. Moreover, direct access to soundscapes can be obtained by interacting with smart panels that are activated by a user’s smartphone. This approach encourages exploration as soundscape playback is based on the simple gesture of passing a smartphone over a panel. Finally, users can access soundscapes while on the move by conducting a proximity search for available soundscapes.

LYS architecture can be divided into two main elements: a web-based server built over a spatial database and a mobile app developed for Android smartphones. The server exposes several API that allow the mobile app to retrieve the audio content and the related information. The system is the result of a joint effort between the private company *Moovioole srl*, which developed the server, and the research team, which designed the interaction and developed the mobile app. LYS components are described in the following segment.

### Uploading and archiving the sounds

The interface to upload and describe soundscapes is based on standard web technologies. We assume that users are familiar with this kind of interface and that it is not an entry barrier for participating in a campaign. We collected the opinions of approximately 170 users who contributed at least one soundscape, and all found the interface quite intuitive.

Given the interest in archiving, LYS allows the user to upload a number of uncompressed audio formats, including monophonic, stereo, binaural, and even advanced formats such as 5.0, 5.1 and Ambisonic. In short, all common sampling rates are possible, although 44.1 kHz or higher is recommended. Most users will likely upload monophonic audio files recorded with their smartphones, but professional users may take advantage of this feature. To this end, it is also possible to archive the information concerning the audio setup, the type of microphone and the recording equipment. The system automatically analyses the audio files and extracts all the metadata that are already embedded. Files are saved in a cloud-based storage system with a compressed version, in MP3 format, which is created on the fly and immediately available on the platform. The choice of storing uncompressed audio, which may require ten times the space of a lossy format, is in accordance with the guidelines for audio archiving. This attention to audio quality, which is a unique aspect of LYS and a few other systems such as *Freesound*, posed problems to about 10*%* of contributors who had no prior knowledge of audio formats. We plan to improve the interface to make it able to deduce the format automatically.

Once the audio file is uploaded and checked, the interface requires the user to provide a complete description of the environment. The user-friendly interface is divided in several sections

- **Spatiotemporal information**: geolocation is based on Google Maps services; in most cases, time is automatically extracted from the audio file.
- **Setting**: either indoor or outdoor.
- **Meteorology**: described through a set of radio buttons, the weather, the kind of wind, precipitation, and presence of water.
- **Environment**: can be selected in different nuances from natural to urban, adding information about various human, machine, or animal elements. The choice can be made using a set of radio buttons labeled by intuitive icons.
- **Narrative**: a short and long narrative describing the soundscape, the occasion of recording, and any other information that is useful for archiving and searching the content.
- **Image**: an image is always associated with the soundscape; if the user does not provide a picture, the system automatically uses a satellite photo.

After the user has provided the relevant information, the record is stored in a MySQL database. A subset of the information is also added to the original audio files as embedded metadata using BWF MetaEdit, an open-source software that supports the specifications of the Federal Agency Digital Guidelines Initiative for audio archiving.

LYS architecture is relatively standard. The framework is a multivendor system based on a customized version Joomla 3 with extensions to manage audio processing libraries. All software modules are written in PHP 7.4 and JavaScript. LYS exploits the services of TOP-IX, an Italian consortium that manages an Internet Exchange infrastructure. Apart from connectivity, TOP-IX provides LYS with two physical machines running Linux CentOS, one for the web portal and the other for managing the audio files. Each time a new user uploads his/her first soundscape, LYS creates a virtual storage on a dedicated NAS.

It is important to note that the web platform’s initial version was designed for sound engineers working in the film industry. Thus, besides providing a rich set of metadata, it offers a number of advanced functions that allow the user to create a personal library of recordings, upload a rich set of audio formats and carry out online audio post-processing.

### Searching and browsing the Web interface

**Fig. 2:**
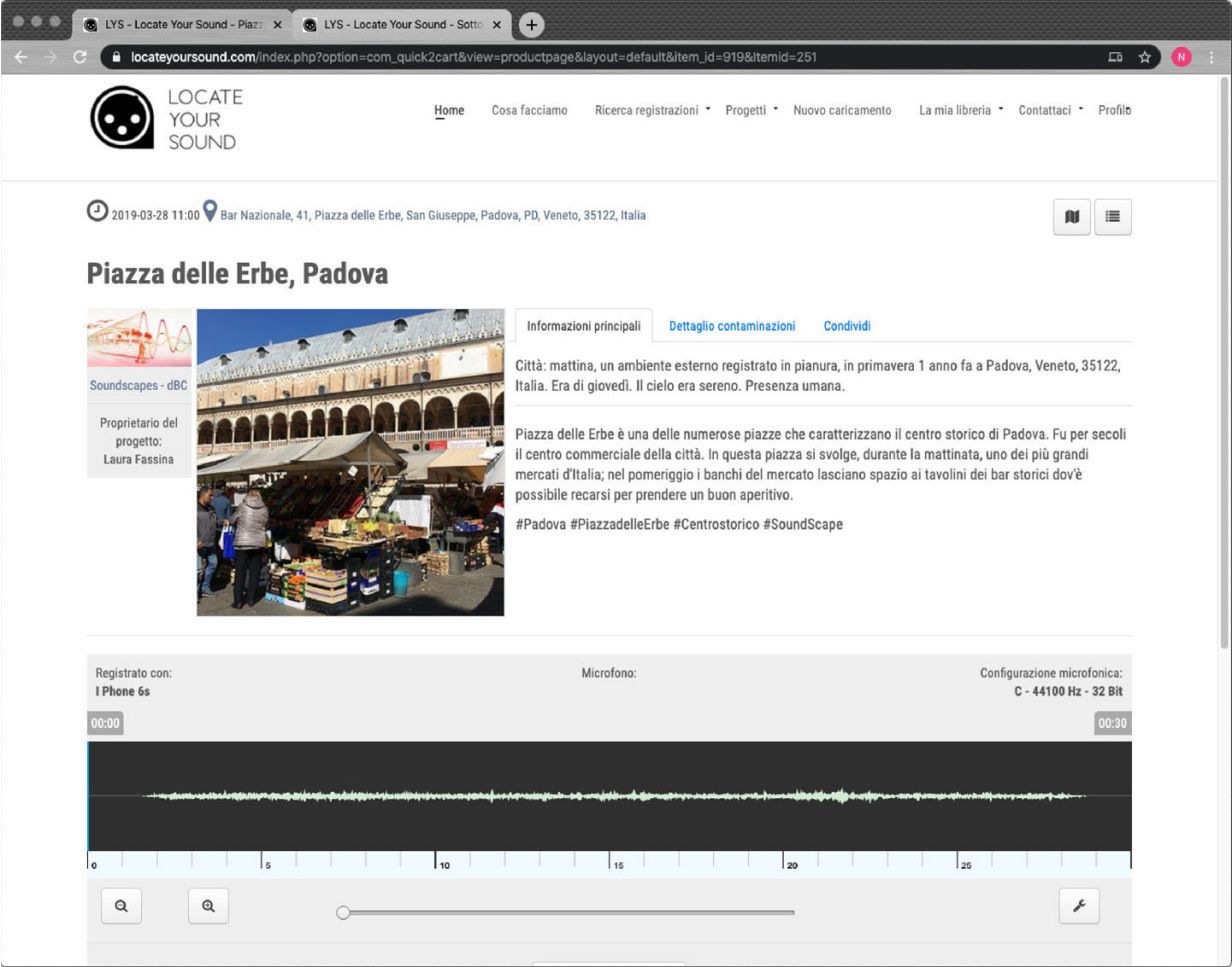
LYS web interface showing the complete records of a given soundscape and the simple audio player representing the soundwave

The straightforward way to access a digital archive is through a web interface. To this end, the LYS web interface provides two standard methods for content access: an interactive geographic map and a text-based faceted search engine. It is possible to filter soundscapes by selecting a campaign, a library, or a single user in both cases. Retrieved soundscapes are shown with the available information in the form of a short narrative with a picture of the place and a simple audio player that depicts the soundwave. The audio is a lossy version in MP3 format of the original audio uploaded by a contributor, and might be post-processed with a compressor, a limiter and a gain controller. Figure 2 shows an example of how the web interface displays a soundscape.

**Fig. 3:**
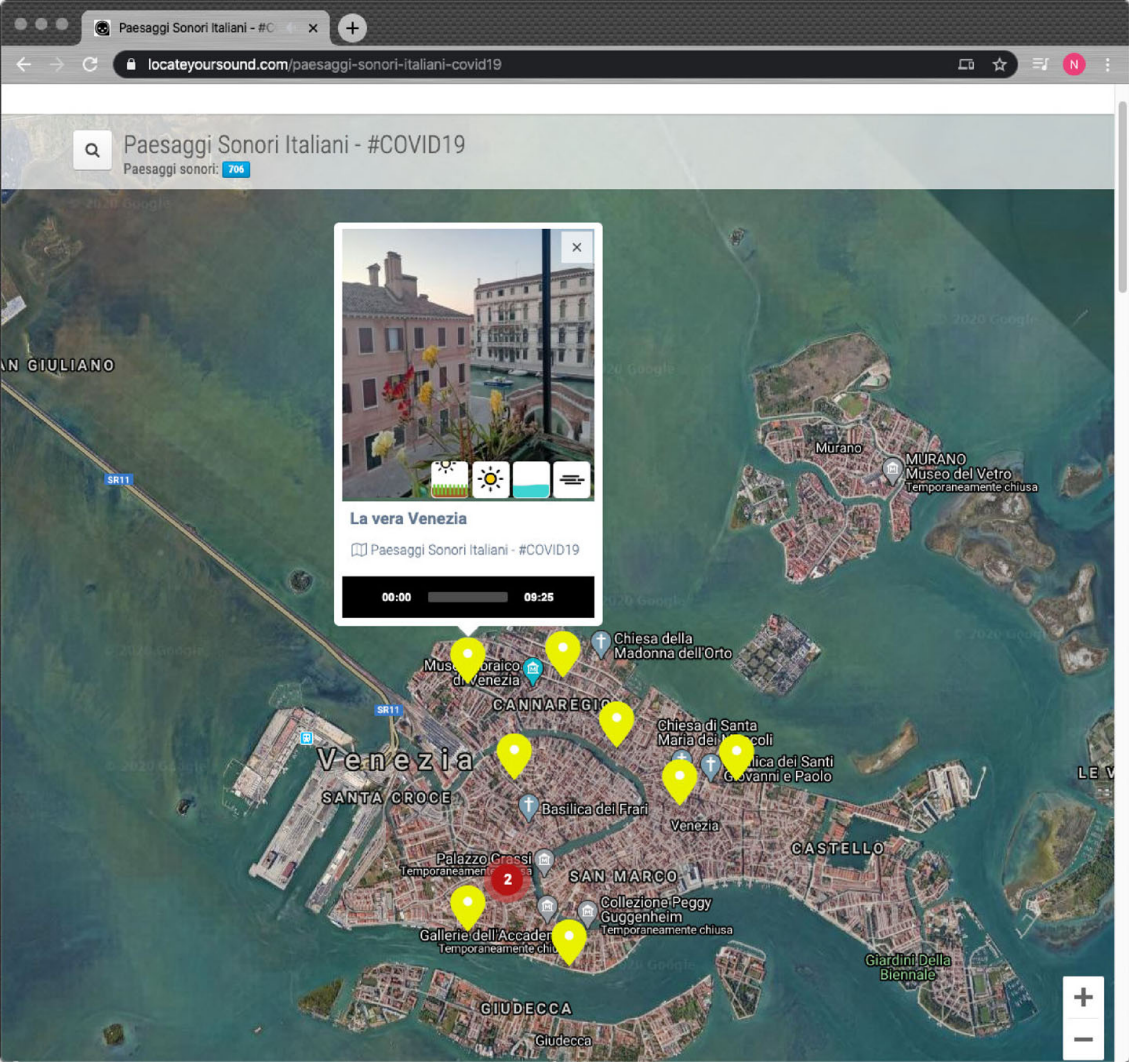
Preview of a soundscape in Venice while browsing on the interactive map

LYS allows for the querying of the geographic database and supports a full-text search on all textual metadata. It is combined with a faceted search on all the available fields – from audio quality to weather conditions – and with the possibility to select soundscapes based on location and time of recording. The browsing function gives a preview of the chosen soundscape, showing the related picture with icons representing the most relevant fields and a simple audio player. As expected, log analysis shows that users generally prefer to use the browsing function rather than the faceted search. An example of a preview is shown in Fig. 3.

### Interacting with smart panels

An additional way to browse soundscapes is through a smart panel and a mobile app. To the best of our knowledge, this is the first project in which the combination of interactive panels and smartphones are exploited to access soundscapes. We believe this form of interaction may be useful for tourists – seeing as the panels would be available at tourist offices, stations, and so on – and for the locals who want to discover hidden aspects of the place they live. A panel can depict the map of a city center or tourist areas, but the scale may vary depending on the goals, so we developed panels of large geographical areas. So far, we have produced smart panels of two city centers, namely the cities of Padua and Vicenza in Italy and the Veneto Region.

The interaction has been designed to highlight the relationship between places and sounds; the experience can be carried out almost without using the smartphone touchscreen or paying attention to the screen itself. The basic idea is that the user simply has to pass his or her smartphone over the panel. Each time the smartphone passes over an active area it vibrates for a few milliseconds and then starts the soundscape. This approach allows for a very gentle learning curve to use the app, as simple as passing an auditory *magnifying glass* over a place. Simplicity is particularly relevant in a tourism scenario, because users have little time to understand how to use an app and need to concentrate on the content. Finally, we believe this form of interaction promotes serendipity because users cannot predict a given place’s soundscape.

**Fig. 4:**
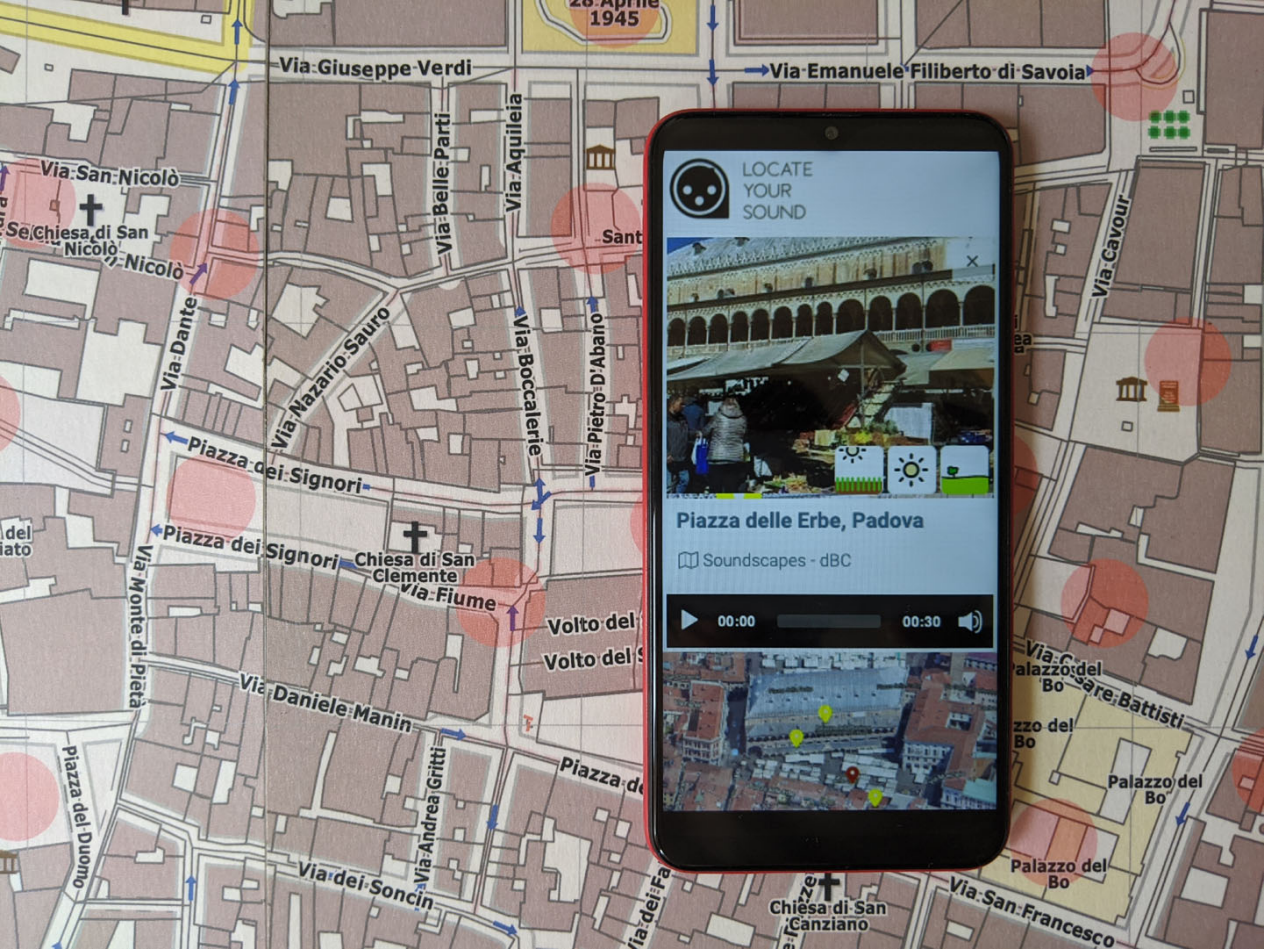
A picture of the smart panel on Padua with the app when a soundscape is found

Although the interaction is mainly based on the auditory and the motor channels, the app also provides visual feedback by showing a picture of the place. Moreover, as the same location is usually associated with multiple soundscapes – recorded in different conditions, at different times of the day or seasons – the user has to preselect the time span of interest. The interactive panel of Padua while a smartphone runs the app is shown in Fig. 4.

Communication between the smartphone and the panels is obtained through Near-Field Communication (NFC) tags linked to geolocated soundscapes through their IDs and hidden behind the panel in the corresponding position in the map. It is worth noting that NFC tags are passive circuits – and thus do not need a power supply – that store a limited amount of information in the order of hundreds of bytes. When an active NFC device, such as one mounted in most smartphone models, comes in a 2 − 3 centimeter range of a passive NFC tag it provides enough energy to activate the passive circuit and communication occurs. In essence, the NFC tag sends the smartphone the information stored in its internal memory – in our case, the set of soundscape identifiers in the LYS database. The technology is relatively inexpensive – an NFC tag costs about fifty cents – and tags are resistant to changes in temperature and humidity, so it is affordable to create several copies of a given panel and distribute them in different locations, even outdoors.

In order to improve usability, and because NFC tags can interfere when they are too close to one another, we carefully selected a subset of locations stored in the LYS database to obtain a homogeneous sampling of the area. Moreover, two graphical versions of the panels were available: a pure map and a map enriched with graphical cues over the NFC tags in the form of red dots. The former provided a higher *wow effect* as participants were not expecting the discovery of a new soundscape location. The latter was easier to interact with, and participants seemed to prefer this version because they could tell how many active points were available and that they were not missing any soundscapes. In both cases, after discovering a soundscape, participants could obtain additional information about the location, its tourist attractions, and its cultural heritage. We carried out a preliminary evaluation by asking 50 participants to interact with two identical panels, apart from the presence of red dots. Slightly more than 80*%* of participants expressed a clear preference for the panels with visual cues, so these were then used for the main evaluation.

The app has been developed in Java for Android smartphones, with Android 4.4 (KitKat) as the minimum SDK due to a major change in dealing with NFC communication. Since KitKat is quite old, we believe this is not a real limitation. Software testing has been carried out on a variety of devices running different Android versions, up to and including Android Pie. NFC tags were type-A with a memory size of 48 bytes that encoded the unique ID of the soundscape stored in the database.

### Accessing soundscapes in mobility

A third way to access soundscapes is through audio augmented reality. As described in Section 3, audio augmented reality has been exploited in several projects aimed at evoking an environment of the past through a synthetic soundscape, usually played through the visitor’s smartphone. Instead, our project aims to provide the user with real soundscapes recorded in the area around his/her location. It might seem counter-intuitive to let visitors listen to recorded soundscapes when they can listen to the one they are presently inhabiting. However, as mentioned in Section 2, the acoustic environment may change dramatically over time. For instance, an off-season tourist might be on a beach usually crowded during summer and perceive the striking contrast with the current quiet and solitude if s/he can hear its summer soundscape. Provided that his/her headphones can reduce the external noise, the reverse can apply as well. A second issue with using real soundscapes is that they might not cover many locations. For this reason, we launched two crowdsourcing initiatives, which are described in Section 5.

**Fig. 5:**
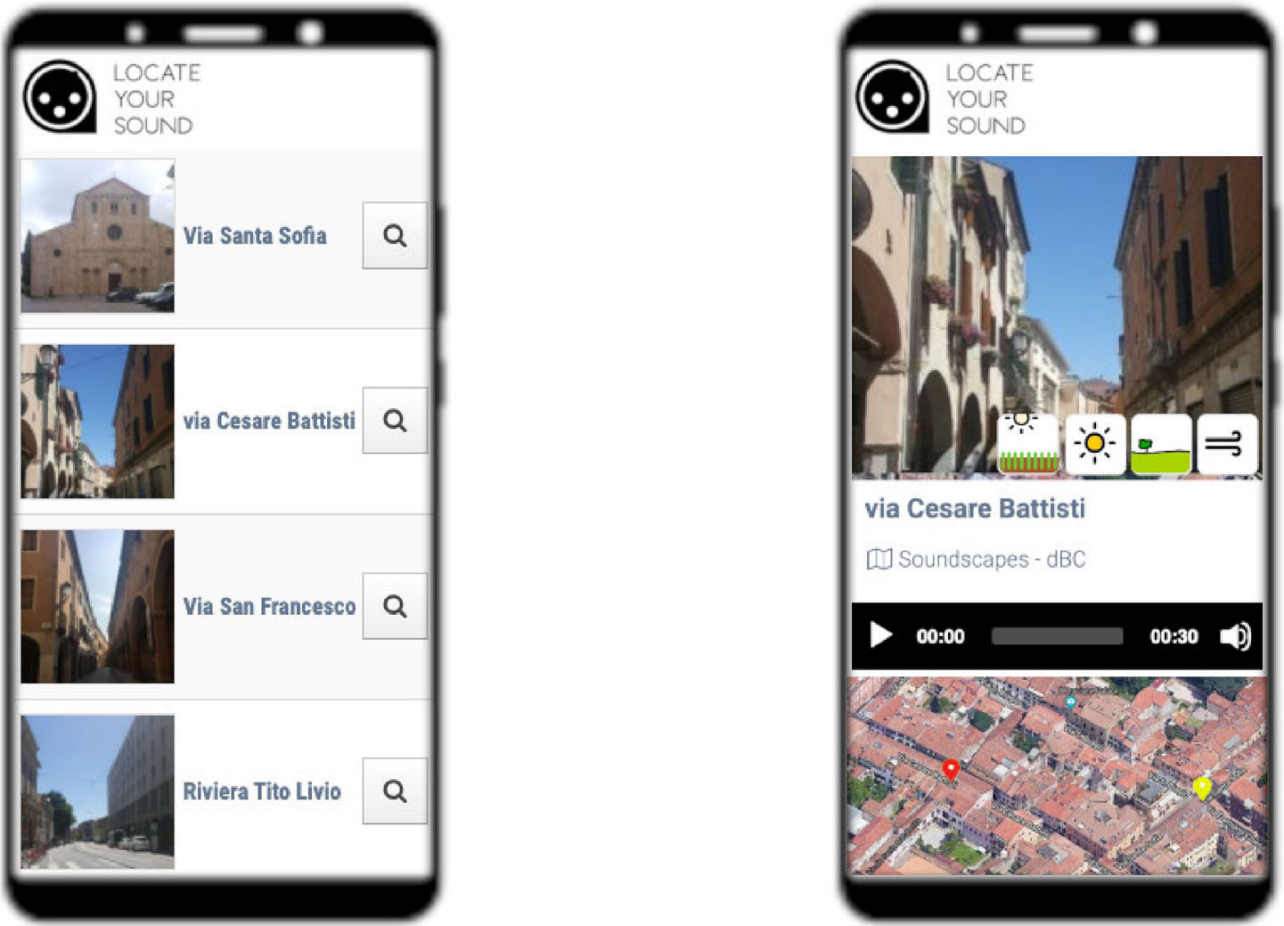
The app screen showing the list of available soundscapes around the user location (left) and a given soundscape after it has been selected (right)

We addressed soundscape access in mobility by including a geolocation module in the Android app that runs as a separate activity. The user can send a range query to the LYS server and obtain a list of available soundscapes ordered by distance to his/her actual location. A given position might have different soundscapes recorded at other times. After selecting the soundscape, the user is presented with the same interface used for smart panels. An example of the list view and the soundscape view is shown in Fig. 5.

Here again the main goal has been simplicity. The user has only to launch the range query, browse a list with minimal information – name and picture of the location – and listen to the soundscape. The graphic design for a given soundscape is identical to the smart panel version as users can exploit both approaches. For instance, a visitor who arrives in the city center can interact with smart panels available at the tourist office and then wander the city and listen to the sounds with whom s/he is already familiar. We can envisage that an acoustic preview can help the tourist identify places. Extensive testing of this hypothesis is planned for future work.

## Crowdsourcing initiatives

As mentioned in Section 4, the Locate Your Sound architecture allows us to run different *projects* that group soundscapes together. Participants can sign up for any project and decide to assign a new soundscape either to a project or his/her personal library. Two of these projects are the result of two crowdsourcing initiatives: *Padua Soundscapes* and *Italian Soundscapes – #COVID19*.

### Padua Soundscapes

The first crowdsourcing experiment involved 47 students attending the undergraduate course in *Design and Management of Cultural Tourism* at the University of Padua. The goal was to collect the soundscapes of the city of Padua, in Northern Italy. Participation was voluntary. As an incentive, depending on their activity, students received up to two bonus points to be added to the final grade of the class in Computer Methods for the Organization of Tourism Services. Although only open to students, the approach shares several characteristics with standard internet-based crowdsourcing methods: students could decide the amount of content to upload, duration and quality were not subject to teacher evaluation, all interaction was carried out using only online resources, and there was no penalty for dropping the project.

**Fig. 6:**
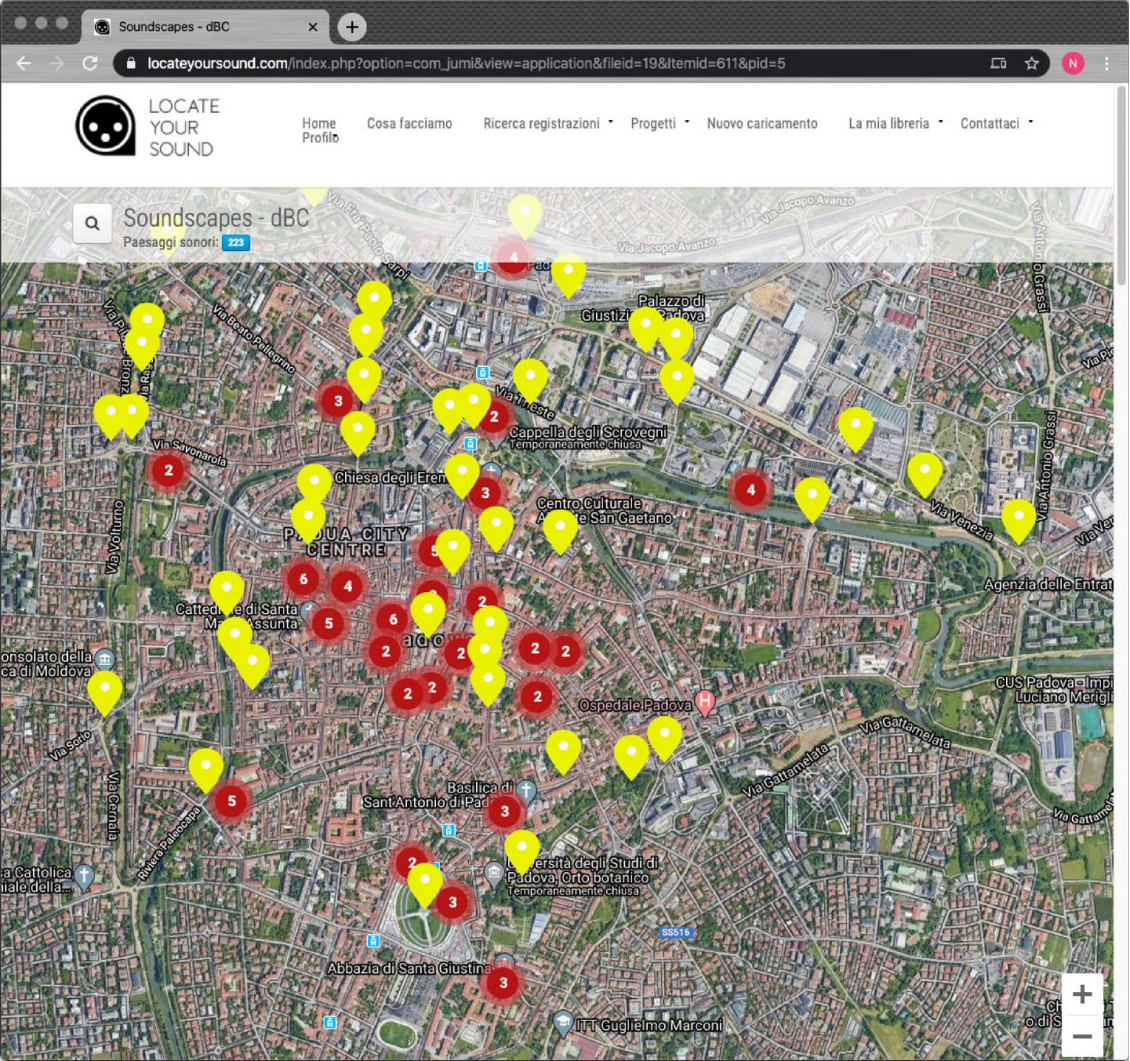
A map of the soundscape recordings produced during the crowdsourcing initiative *Padua Soundscapes*

When a crowdsourcing approach is undertaken, certain crucial aspects must be taken into account. The simple motivation to participate, which here was assured by the possibility of improving one’s mark, does not guarantee effective collaboration among participants and standardized content quality. In order to promote collaboration, the project included a wiki and a forum where participants could propose new locations and schedule recording sessions. Moreover, the wiki became the shared space where participants provided additional contributions to the project. For instance, a group of students proposed acoustic paths across different locations. We plan to include these paths in a future version of the mobile app. Notwithstanding these positive results and collaboration tools, the selection of locations was skewed: some cultural landmarks were represented, but others (e.g., the train station or the entrance of the Department of Cultural Heritage that hosts the course) were likely chosen due to their ease of reach. The campaign started in March 2019 and lasted until July 2019. Figure 6 shows the LYS interface where all the 223 soundscape recordings are shown; depending on the zoom level, round red dots represent soundscape groups (their number is reported within the circle). These results suggest the feasibility of a crowdsourcing approach, at least for a city the size of Padua.

The audio material used in the smart panels described in Section 4.3 has been selected during the development of a M.S. thesis in Tourism Applications. A parallel experiment has been carried out in Vicenza by a single student for his Master’s thesis, in which recordings were made of a limited number of places at three different times of the day.

### Italian Soundscapes – #COVID19

The second crowdsourcing initiative has been carried out in collaboration with the Central Institute for Sound and Audiovisual Heritage (Istituto Centrale per i Beni Sonori e Audiovisivi – ICBSA), a public institute responsible for documenting, promoting, and preserving national sound and audiovisual heritage.

Italy and many other countries were severely affected by the outbreak of the novel coronavirus. Italy was on a nationwide lockdown from early March until early May 2020. At the time of writing, the dramatic social and economic impacts are still difficult to measure and are beyond the scope of this paper. However, what is relevant is how the lockdown drastically changed the Italian soundscape. As everybody quickly noticed, the absence of the hustle and bustle of cars and machinery let subtler sounds emerge, thus creating an entirely new soundscape.

This crowdsourcing initiative aimed to preserve the memory of this new acoustic environment by archiving and making it available to citizens in the future. We launched the call for contributions on March 16, 2020, using the official channels of the Central Institute for Sound and Audiovisual Heritage and the Department of Cultural Heritage of the University of Padua and their profiles on the leading social platforms (YouTube, Facebook, Instagram). The call clearly stated that recordings should be taken in total compliance with the lockdown, hence from windows, balconies and private gardens. Moreover, to increase the number of soundscapes, we asked participants to involve friends in other locations. According to the call, each participant should provide four recordings taken at different times of the day and contact at least ten friends. Participation was continuously encouraged with daily updates on the number of soundscapes and direct contact with audio enthusiasts’ societies.

**Fig. 7:**
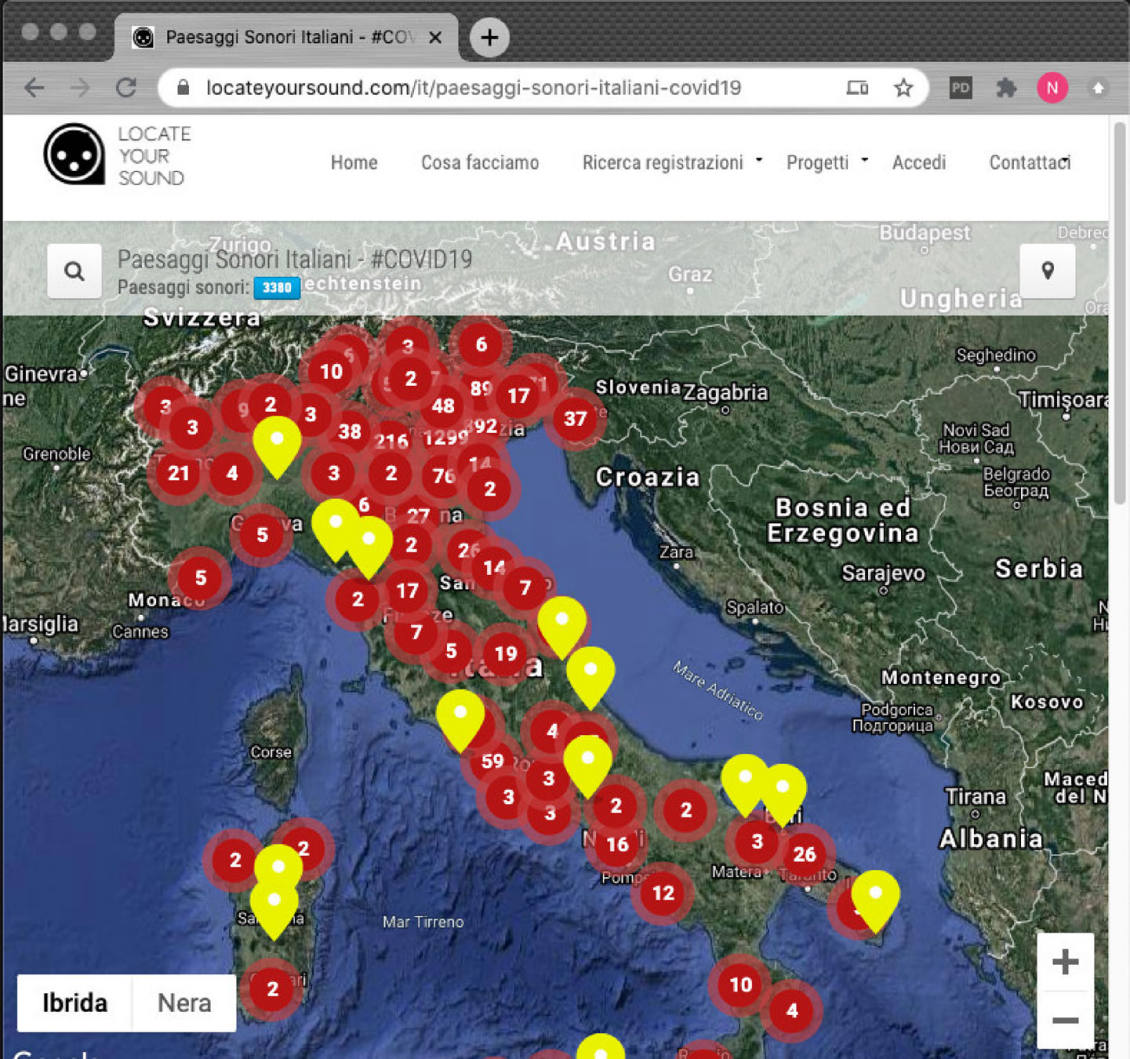
A map of the soundscape recordings produced during the crowdsourcing initiative *Italian Soundscapes – #COVID19*

By the end of the lockdown, approximately 200 participants uploaded 3370 Italian soundscapes (plus four European and six South-American soundscapes), mainly from Northern Italy, which was the area most struck by COVID-19 at the time. Several hundred hours of soundscapes are now stored in LYS and available for listening. The material will be the basis for the creation of smart panels for different cities, allowing for the exploration of an urban environment absent of human-generated sounds. The material will also be used in the mobile version of the app when the pandemic is over. Figure 7 shows the LYS interface where the soundscape recordings are shown.

It is interesting to analyze how Italy was mapped during the campaign. Although we asked participants to record the soundscapes outside their homes (in essence what can be heard from their windows), 12*%* of the recordings are of indoor environments. Participants likely wished to underscore how forcing a nation to live inside their home also transformed internal soundscapes. The type of location is evenly distributed among cities, suburbs, and countryside, while there are differentiations in the time of day – with 53*%* of soundscapes recorded in the afternoon and only 15*%* recorded at night – and on the weather conditions – with 6*%* recorded during rain – although this latter outcome is probably influenced by the exceptionally sunny weather during the lockdown.

## Evaluation

We performed out a qualitative evaluation of how users interact with LYS. In particular, we addressed the usage of smart panels and made a quantitative evaluation of the effectiveness of the crowdsourcing campaigns.

### Smart panels for padua soundscapes

Our goal was to assess interactive smart panels’ capability to engage users and raise interest in the locations. In particular, we aimed at evaluating the point of view of final users who were unaware of soundscape projects and could choose this experience among other competing offers. To this end, tests were carried out during public dissemination events organized in Padua, namely *CHItaly* on September 24, 2019, *Researchers’ Night* on September 27, 2019, and *DigitalMeet* on October 24, 2019. As mentioned in Section 4.3, smart panels depicted the city centers of Padua and Vicenza with soundscapes gathered through the Padua Soundscapes campaign. We also tested the web interface, which provided standard interaction.

Due to difficulties in gathering quantitative data during public events, we carried out a qualitative study using the *think-aloud* protocol [4] by asking participants to use the system while continuously verbalizing their thoughts. Thinking aloud was facilitated by the fact that participants usually attend these events in small groups of two or three people and naturally share their impressions with friends while checking the available systems.

The assignment was straightforward: discover soundscapes, listen to them for an unrestricted amount of time (possibly shorter than soundscape duration), and read the associated information if there was in interest. Instructions were given orally to each participant, using wording that was previously agreed. Moreover, participants were invited to perform simple tasks.

- Find a quiet place to relax and read a book.
- Choose where to go for a drink in a lively neighborhood.
- Identify streets affected by traffic jams.

About one hundred participants took part in the tests. We did not ask for their ages, but most were young adults from 20 to 35 years old. Children also interacted with the interfaces, but their impressions were not tracked. Gender was quite equally distributed, with 60*%* female and 40*%* male. One-third of the participants tested the web interface – which was less appealing – and the rest tested the smart panels. Soundscapes could be heard using computer loudspeakers for the web interface and smartphone speakers for the smart panels. The surrounding acoustic environment – i.e., the soundscape around the experimental area – did not interfere with the playback of recorded soundscapes.

Overall, results were largely positive with some differences between the two interaction modalities. Due to the widespread usage of geolocation interfaces, the web interface was considered quite intuitive. The majority of participants expressed their intention to listen to places they already knew or to city landmarks, expressing the wish to confirm their memories or their expectations. To this end, they stated they would like to listen to the soundscapes directly without having to open a pop-up with the player (see Figure 4.2). There was a moderate interest towards the pictures of the location, while a limited number of participants expressed an intention to read the text information, which required an additional click. Some participants used the system to discover the soundscape of unknown locations, expressing their curiosity verbally. For the city of Padua, the most common sentence in this case was akin to “There are cars everywhere!”, which was a way to manifest an enhanced awareness of urban soundscapes.

In terms of the smart panels, which displayed the city centers of Padua and Vicenza, the idea of using a smartphone as a medium to interact with multiple locations was appreciated by participants. As most of them stated, people are becoming used to contactless communication through smartphones. That being said, this usually occurs in a point-to-point interaction. The smart panels were novel enough to be interesting but sufficiently familiar to be usable. Some participants expressed initial trepidation about interacting with the panel, wondering aloud what they were supposed to do. Moreover, a large number preferred to use the smartphone provided by the experimenter rather than their own. Once initial resistance was overcome, participants enjoyed the experience, stating that the approach could be useful for tourism applications as a tool to experience a location before actually visiting it.

Exploration was the main activity with smart panels; the app automatically plays a new soundscape – with a picture of the location as shown in Fig. 4 – as soon as a new NFC tag enters the smartphone range. Since the city map panel showed a red dot over each NFC tag, some participants checked all the soundscapes in a systematic order, while others moved randomly over the panel. Few participants verbalized their intention to check a particular place they knew, likely because the city map covered only the historical center. It is unsurprising that in the case of smart panels, too, local participants were pleased to recognize their city’s sounds and surprised by the large amount of machine-related sounds.

As mentioned, interacting with the web interface encouraged studies or visits to known locations while interacting with smart panels promoted exploration. This may be due to the size of the area covered by the experience as smart panels have obvious physical limitations. It may also be caused by the simplicity of interaction in moving an object over a panel. This difference also had an impact on the duration of the soundscape playback, which was longer with the web interface. On the contrary, the coverage was higher with the smart panels and, in general, the experience lasted longer.

### Participation to italian soundscapes – #COVID 19

Our goal was to assess whether the use of LYS and participation in the crowdsourcing campaign affected how users perceive soundscapes and their interest in accessing recording provided by others, whether friends or strangers. Moreover, another research question regarded the long-term effects of actively participating in a campaign in terms of awareness of the acoustic environment and intention to keep on recording soundscapes.

Through online questionnaires, assessment was carried out using a 5-level Likert scale, from *totally disagree* to *totally agree*. Among the 200 participants of the crowdsourcing campaign, 50 subjects answered the questionnaire, which was available at the beginning of September 2020 for about a week. Subjects were mainly female (78*%*), ranging from 19 to 55 years (average 24.2 years).

In terms of the questions about active participation, subjects enjoyed providing sounds. Nobody disagreed with the sentence *“Sound recording was an intriguing experience”*, which obtained an average score of 4, with 30*%* subjects in total and 40*%* in partial agreement; the remaining 30*%* were neutral. On the contrary, poor results were given to system usability for uploading and describing the files; the sentence *“LYS was simple to use”* obtained an average score of 2.8 with 36*%* of subjects in disagreement and 36*%* neutral. This outcome will be taken into account for future developments of LYS.

**Fig. 8:**
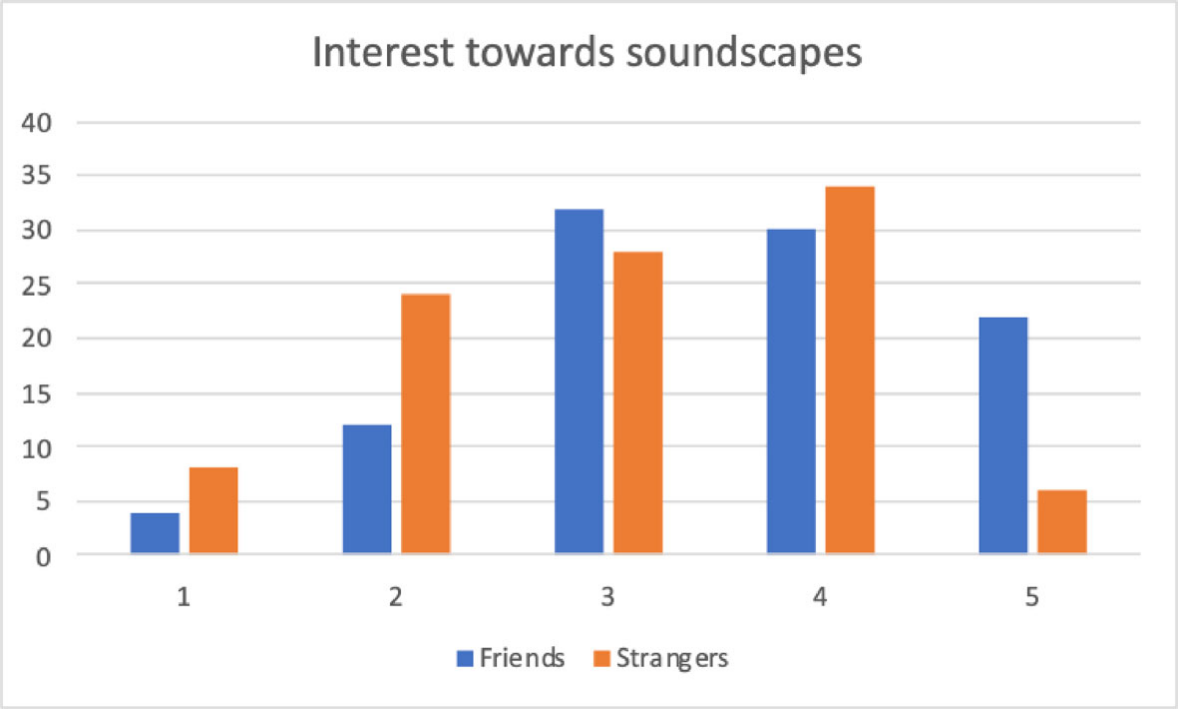
Comparison of subjects’ interest towards soundscapes provided by their friends or by strangers (1 = no interest; 5 = total interest)

Another important aspect regards interest in accessing soundscapes, which was investigated by two sentences: *“I listened with interest to soundscapes provided by friends”* and *“I listened with interest to soundscapes provided by strangers”*. Figure 8 compares the agreement to these two sentences, which obtained an average score of 3.54 and 3.06 respectively. It seems that recording and sharing soundscapes has a social component that plays an important role in increasing user interest.

The sentence *“I realized how much the acoustic environment changed during lockdown”* investigated the impact of the crowdsourcing campaign in raising participant awareness. Results were quite high, with 48*%* in total agreement and 28*%* in partial agreement (only three subjects partially disagreed and the rest were neutral); the average score was 4.18. Thus, active participation in tracking an event plays an important role in raising awareness in the scope of the event itself.

**Fig. 9:**
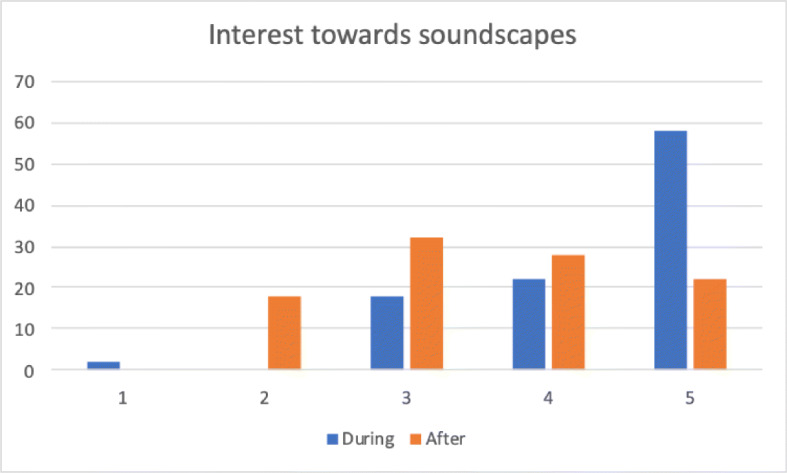
Comparison of subjects’ interest towards soundscapes during and after the campaign (1 = no interest; 5 = total interest)

We also wanted to assess whether contributing to LYS had long terms effects on user perception of the acoustic environment. This was investigated through two similar sentences: *“During the campaign I paid significant attention to the acoustic environment”* and *“I am still playing more attention to the acoustic environment”*. Figure 9 compares the agreement to these two sentences, which obtained an average score of 4.34 and 3.54. Although there was a clear drop in interest, 50*%* partially (28*%*) or totally (22*%*) agreed that participation in the campaign changed their attitude towards soundscapes months after the end of the campaign.

A final sentence, *“I have an interest in recording acoustic environments”* explored the possible influence towards general soundscape recording, with more subject disagreeing and an average score of 2.62.

### Comparison of the campaigns

We gathered additional feedback through a semi-structured discussion with 30 students who participated in the *Padua Soundscape* campaign and through 85 written reports by contributors to *Italian Soundscapes – #COVID*.

There was a general consensus that the crowdsourcing campaigns were useful to increase their awareness about sound’s pervasiveness in any environment. Recalling the distinction in the kind of listening [6], the sounds that were part of holistic listening cross the threshold to become part of attentive listening. Yet, the two groups showed differences in the way they thought that a soundscape was *relevant*.

On the one hand, the vast majority of the *Padua Soundscape* group felt that soundscapes were interesting only if something happened during the recording. Soundscapes not telling a story were perceived as uninteresting, and thus participants tried to capture variations in the acoustic environment rather than steady sounds. Moreover, they mentioned soundscape *aesthetics*: a soundscape should be pleasant rather than informative. It is worth recalling that participants were enrolled in an undergraduate course in Design and Management of Cultural Tourism. Hence, they were interested in using soundscapes to promote a location.

On the other hand, a large part of the *Italian Soundscapes – #COVID* group found the presence of steady or continuous sounds more relevant. There was no need to tell a story, because a larger narrative already existed: the lockdown. In terms of the pleasantness of soundscapes, most reports highlighted that the discovery of previously unnoticed sound layers was pleasant in itself. Additionally, most participants expressed how recording the changed environment helped them realize the effects of the lockdown.

## Conclusions

This paper describes a project about archiving, browsing, and accessing soundscapes, either remotely or on-site, through a mobile app. A complete system, called *Locate Your Sound* has been developed. The two main components are a web-based server built over a spatial database and a mobile app. Three different approaches to accessing the geolocated soundscapes are proposed: browsing and searching a web interface, interacting with smart panels via a smartphone app, and accessing the collection on the move using smartphone GPS. The system is available to users who contribute new soundscapes or wish to access existing ones. We launched two crowdsourcing initiatives to populate LYS. The first initiative involved students at the University of Padua in mapping the sounds of the city. The second aimed at tracking how the Italian lockdown for COVID19 affected the acoustic environment.

Results are encouraging. The audio channel has unexplored potential, notwithstanding the increasing number projects about soundscapes. LYS aims at both professional and common users. It allows for high quality audio and provides post-processing audio tools to improve the listening experience. It also focuses on archiving intangible cultural heritage. Assessment through the think-aloud protocol showed that, among the different interaction tools developed, the web interface encourages users to compare the recorded soundscapes with their memories and expectations, while smart panels promote exploration.

A quantitative evaluation was carried out using questionnaires. The most relevant outcomes are that active participation in LYS campaigns can increase user awareness of the acoustic environment, which is particularly high during the campaign and persists after months. Access to soundscapes is also influenced by social aspects, as friends’ recordings seem to be more interesting than those provided by strangers.

Future work will focus on developing and testing the audio augmented reality approach based on user geolocation. The user interface can be improved by allowing the user to easily select a particular recording for a given location. The actual version simply lists all the available recordings without grouping them according to location. Extensive testing will be carried out once tourism in Italy recovers from the effects of the pandemic. Another research direction will be the use of the collected material to train automatic sound source classification. Given the rich amount of descriptive metadata and the size of the collection, it will be possible to develop tools that automatically compute descriptive metadata to speed up the archiving process.

We plan to launch additional crowdsourcing campaigns. In particular, we are participating in an Italian project on sound memories, where users are invited to share audio recordings – possibly from home videos – from their private collections. The campaign is promoted by the Central Institute for Sound and Audiovisual Heritage, which was a partner for the second crowdsourcing initiative.
